# Implementation and outcomes of a multifaceted care model for people experiencing homelessness and people who inject drugs hospitalized with complicated infections

**DOI:** 10.1017/ash.2026.10406

**Published:** 2026-05-11

**Authors:** Geehan Suleyman, Dana M. Parke, Simran Brar, Caren El-Khoury, Seema Joshi, Phina Ross, Lynsey Halpin, Rachel M. Kenney, Michael P. Veve

**Affiliations:** 1 Henry Ford Health System, USA; 2 Wayne State University School of Medicine, USA; 3 Wayne State University Eugene Applebaum College of Pharmacy and Health Sciences, USA

## Abstract

**Background:**

People experiencing homelessness (PEH) and people who inject drugs (PWID) face disproportionate burdens of infection and poor clinical outcomes due to social determinants of health (SDOH) barriers such as unstable housing, limited medication access, and poor follow-up care. The study purpose was to evaluate the impact of a multi-model quality improvement (QI) bundle on outcomes of hospitalized PEH/PWID.

**Methods:**

A pilot QI initiative was implemented at an urban academic hospital in Detroit (June 2022–April 2023) targeting hospitalized PEH/PWID requiring ≥2 weeks of antimicrobials. Bundled prospective intervention included specialty consultation, tailored antibiotic education, medication cost inquiry, Meds-to-Beds delivery, enhanced case management coordination, and referrals to community-based organizations addressing SDOH needs. Ninety-day clinical outcomes were described.

**Results:**

42 patients were included: 26% PEH, 29% PWID. Patients were predominately men (62%), White (57%), and with a median (IQR) age of 43.5 (32.5–53) years. Common infections included bacteremia (60%), bone/joint infections (50%), and endocarditis (31%); *Staphylococcus aureus* was the leading pathogen. Over half of patients were discharged on oral antibiotics. Treatment adherence remained low: 29% self-discharged, 52% were unreachable postdischarge, and 21% attended scheduled follow-up. 43% of patients achieved clinical cure and 38% experienced disease progression within 90 days. Emergency department revisits (29%) and hospital readmissions (43%) were frequent.

**Conclusions:**

Despite a multi-model QI intervention, hospitalized PEH/PWID with complicated infections experienced significant barriers to care, resulting in poor adherence and outcomes. Pragmatic, scalable interventions and community partnerships are needed to improve health outcomes.

## Background

In the United States, an estimated 3.7 million individuals inject drugs and approximately 650,000 people experience homelessness (PEH).^1,2^ These populations face a disproportionate burden of infectious diseases and worse health outcomes compared to the general population.^3–7^ People who inject drugs (PWID) and PEH are hospitalized more frequently, experience prolonged hospital stays due to discharge barriers, and often struggle with medication adherence, contributing to suboptimal clinical outcomes.^3^ PWID also account for approximately 7% of new HIV infections annually.^8^

The care of PWID and PEH is further complicated by numerous social determinants of health (SDOH), including unstable housing, lack of transportation, limited financial resources, and insufficient secure storage for medications.^3, 9–11^ These barriers not only hinder effective treatment of infectious diseases but may also contribute to the development of antimicrobial resistance.^11–15^ Up to 80% of an individual’s health is influenced by SDOH,^16^ and existing literature suggests that Black, Hispanic, and low-income populations are at increased risk for community-acquired antimicrobial resistance, including methicillin-resistant *Staphylococcus aureus.* ^14^ Outpatient parenteral antimicrobial therapy (OPAT) is often indicated for prolonged treatment of complicated infections.^3^ However, access to OPAT requires stable housing, reliable transportation, and admission to facilities such as skilled nursing facilities that facilitate intravenous antibiotic administration.^3^ These requirements frequently exclude PEH and PWID from eligibility. For PWID, additional concerns such as catheter misuse, increased risk of central line-associated infections, and high rates of self-directed discharge further limit OPAT eligibility.^3,17,18^

Stigma plays a critical role in limiting access to care and adherence to treatment for PEH and PWID.^19–22^ Both perceived and experienced discrimination can discourage individuals from seeking medical attention, while internalized stigma may further hinder engagement with essential health services.^19–23^ Access to medication for opioid use disorder and psychiatric care is often constrained by financial hardship, limited availability of treatment programs, and restrictive policies.^23–28^ These barriers not only obstruct access to care but also undermine treatment adherence,^23,29^ making it difficult for individuals to maintain continuity in their care. Although integrated models that address both mental health and substance use disorders (SUD) are essential,^30,31^ their implementation remains limited due to lack of insurance coverage, transportation challenges, and insufficient mental health infrastructure.

Several innovative interventions have demonstrated success in improving outcomes for PWID and PEH.^22,32,33^ These include Tele-Harm Reduction, which leverages telehealth to reduce stigma and improve access to care for PWID with HIV^22^; nurse-led outreach models that deliver hepatitis C treatment via mobile units^32^; and integrated mobile services that provide medication for opioid use disorder and HIV care in community settings.^33^ Despite these advancements, targeted interventions for PEH and PWID with complex infections remain limited. To address this gap, the study team launched a quality improvement (QI) initiative at our institution aimed at advancing health equity and improving clinical outcomes for hospitalized PEH and PWID who require prolonged antibiotic treatment. The project focused on identifying key SDOH barriers and implementing targeted, multidisciplinary interventions to enhance care delivery, support treatment adherence, and strengthen postdischarge continuity.

## Methods

### Study design and participants

This pilot QI initiative was conducted at Henry Ford Hospital, an 877-bed academic tertiary care center in Detroit, Michigan, from June 2022 to May 2023. The study evaluated a QI initiated focused on hospitalized adults who were PEH or PWID with complicated infections (eg, bone/joint infections, infective endocarditis) requiring a minimum of 2 weeks of antimicrobial therapy. Patients discharged prior to multidisciplinary team intervention or who died before hospital discharge were excluded. The primary endpoints were clinical cure and 90-day all-cause and infection-related readmissions. Secondary outcomes included ambulatory case management (ACM) follow-up, infectious diseases appointment attendance, number of emergency department (ED) visits, and treatment completion. The study was approved by the Henry Ford Health Institutional Review Board.

### Patient identification

Eligible patients were identified during weekday working hours by the interdisciplinary study team. The preintervention study period included developing recruitment strategies, such as the creation and dissemination of informational fliers to Henry Ford Hospital providers, pharmacists, nurses, and case managers, who were instructed to notify the study team via secure messaging when a potentially eligible patient was identified. This was supplemented by electronic health record (EHR) review using Epic

SlicerDicer (Epic Systems Corporation, Verona, WI), which screened for PEH or PWID with infectious admitting diagnoses based on International Classification of Disease, Tenth Revision, codes (via billing, encounter, or hospital problem list). Inclusion criteria required receipt of at least 1 antimicrobial during hospitalization and documentation of either an active or historical diagnosis of opioid-related disorders (F11.XX) or homelessness (Z59.XX). Daily eligibility assessments were also conducted during internal medicine progression rounds.

### Study interventions

The interdisciplinary team, comprising infectious diseases, clinical pharmacy, population health, and case management, met regularly to plan and implement a multi-modal intervention structured around 4 core components:

1. **Discharge Antimicrobial Planning and Medication Access**
  - Developed tailored educational materials and guidance document for providers on antimicrobial selection considerations for PWID and PEH (ie, storage requirements for patients without refrigeration, antimicrobial selection given patient specific factors).
  - Integrated a SDOH screening wheel assessment into the pharmacy view of the EHR in November 2022.
  - Refined the discharge medication cost inquiry consult order in the EHR to improve documentation of medication access concerns and cost-related barriers.
  - Leveraged the pharmacy’s Meds-to-Beds Program to facilitate in-hospital delivery of oral antibiotics prior to discharge. This program includes delivery of discharge medications directly to the patient’s hospital room predischarge.
2. **Enhanced Case Management Coordination**
  - Strengthened workflows between inpatient case management and ACM to ensure warm handoffs and continuity of care for high-risk patients prone to loss to follow-up.
3. **Community-Based Referrals and Outreach**
  - Facilitated referrals to community-based organizations addressing housing, food insecurity, transportation, employment, and other SDOH needs.
  - Integrated the Street Medicine Detroit consult order into the EHR in September 2022, enabling in-hospital visits by volunteer medical students to coordinate postdischarge outreach for PEH.
4. **Substance Use Disorder Management**
  - Utilized addiction medicine consult orders to provide comprehensive assessment and management of SUD for PWID during hospitalization.

After intervention implementation, the study team met at least monthly to discuss patient cases and outcomes, and opportunities for improving the intervention process. Barriers and enablers were identified using study team member consensus for both patient populations throughout the intervention period.

### Key definitions

PEH referred to individuals who reside in shelters, live in transitional housing, or sleep outdoors or in vehicles. PWID included individuals who identified as actively self-injecting drugs in the electronic medical record. Ninety-day all-cause readmission was defined as any hospitalization occurring within 90 days of index discharge. Ninety-day infection-related readmission was defined as any hospitalization within 90 days of index discharge attributable to relapse, progression, or complications of the index infection, including readmissions related to premature self-directed discharge and/or failure to complete prescribed antimicrobial therapy. Clinical cure was defined as resolution of infection-related signs and symptoms without escalation or re-initiation of antimicrobial therapy, procedural or surgical intervention, or infection-related readmission within 90 days of completing antibiotic therapy. Disease progression/relapse was defined as clinical worsening while on appropriate antimicrobial therapy. All-cause, 90-day mortality was defined as death due to any cause within 90 days of the index hospital discharge.

### Data collection

Data were manually collected from the EHR using a standardized electronic case report form. Variables collected included patient demographics, clinical syndrome and microbiologic data, antibiotic treatment, interdisciplinary interventions (ie, specialty consultations), SDOH factors, and clinical outcomes. Specialty consultations included addiction medicine and street medicine; street Medicine consultation includes specialty evaluation of access to quality medical care for unreached and service resistant PEH.

### Statistical analysis

Descriptive statistics were used to summarize patient characteristics and outcomes, including proportions and medians with interquartile ranges. All analyses were performed using IBM SPSS Statistics, version 22.0 (IBM Corp., Armonk, NY).

## Patient consent statement

The study was approved by the Institutional Review Board of HFH, Detroit, Michigan. Informed consent was waived given that the study exclusively used deidentified data.

## Results

Forty-two patients were included in the study (Table 1). Of these, 12 (29%) were PWID, 11 (26%) PEH, and 19 (45%) both; among the PEH, nearly half were unsheltered. The cohort was predominantly male (62%) and White (57%), with a median (IQR) age of 43.5 (32.5–53) years. Twelve (29%) had prior hospitalization within 30 days for the same infection. The most common infections were bacteremia (60%), bone and joint (50%), and endocarditis (31%). Over half of the patients required surgical intervention, and *S. aureus* (55%) was the predominant pathogen. The average (SD) length of stay was 13.4 (±10.4) days.

**Table 1.** Demographics, clinical characteristics and treatment of people who inject drugs (PWID), experience homelessness (PEH), or both (PEH and PWID)

	Total N = 42 (%)	PEH or both N = 30 (%)	PWID N = 12 (%)
Median age, years (IQR)	43.5 (32.5–53)	46 (33.3–56.5)	36 (31.5–50.5)
Male	26 (62)	20 (67)	6 (50)
Race/ethnicity			
White	24 (57)	15 (50)	9 (75)
Black	15 (36)	12 (40)	3 (25)
Chronic hepatitis C virus	14 (33)	7 (23)	7 (58)
HIV	3 (7)	2 (7)	1 (8)
If experiencing homelessness, unsheltered	14 (33)	14 (47)	NA
Prior hospitalization within 30 days	12 (29)	10 (33)	2 (17)
Infection type			
Bacteremia	25 (60)	17 (57)	8 (67)
Bone and joint	21 (50)	17 (57)	4 (33)
Infective endocarditis	13 (31)	7 (23)	6 (50)
Pneumonia with or without empyema	10 (24)	6 (20)	4 (33)
Skin and soft tissue infection	6 (14)	3 (10)	3 (25)
Other	11 (26)	8 (27)	3 (25)
Infectious organism			
**Methicillin-resistant *Staphylococcus aureus***	16 (38)	10 (33)	6 (50)
**Methicillin-susceptible *S. aureus***	7 (17)	4 (13)	2 (17)
*** Streptococcus*** **species**	4 (10)	3 (10)	1 (8)
Enterobacterales	5 (12)	4 (13)	1 (8)
Polymicrobial	6 (14)	4 (13)	2 (17)
No culture	3 (7)	3 (10)	0
Required surgical intervention	25 (60)	18 (60)	7 (58)
Discharge antibiotic route			
Intravenous	10 (24)	7 (23)	3 (25)
Oral	23 (55)	16 (53)	7 (58)
Intravenous and oral	1 (2)	1 (3)	0
No antibiotics^*^	8 (19)	6 (20)	2 (17)
Discharge antibiotic			
* β*-lactam	10 (24)	8 (27)	2 (17)
Trimethoprim/sulfamethoxazole	8 (19)	6 (20)	2 (17)
Dalbavancin	7 (17)	5 (17)	2 (17)
Doxycycline	6 (14)	4 (13)	2 (17)
**Other** ^†^	9 (21)	6 (20)	3 (25)
Average treatment duration, days ± SD	29.3 ± 16.5	29.4 ± 16.5	29.17 ± 17.4
Average length of stay, days ± SD	13.4 ± 10.4	13.5 ± 10.9	13.3 ± 10.4

* One patient completed antibiotic course prior to discharge; 7 had self-directed discharge prior to receiving the antibiotic or prescription. ^†^This included quinolones, daptomycin, and linezolid.

### QI interventions

Discharge medication cost inquiry orders were placed for 11 patients (26.2%), most commonly for oral anticoagulants, inhalers, linezolid, moxifloxacin, trimethoprim/sulfamethoxazole, and buprenorphine/naloxone. More than half of patients were discharged on commonly prescribed oral agents including *β*-lactams (24%), trimethoprim/sulfamethoxazole (19%), dalbavancin (17%), and doxycycline (14%). The average (SD) treatment duration was 29 (±16.5) days. Of the 31 patients eligible for the Meds-to-Beds Program, 15 (48.3%) received their antibiotics prior to discharge. Nine patients (20.1%) had a self-directed discharge before medications were delivered; 2 were given prescriptions, but only 1 picked it up. Among the 7 patients sent to the pharmacy, 5 successfully picked up their medications.

Addiction medicine consult orders were placed for 29 of the 31 PWID (93.5%); 2 patients declined. Street medicine consult orders were placed for 11 of 27 eligible PEH (40.7%) after the resource was introduced in September 2022, including 4 of 9 unsheltered PEH (44.4%).

Integrated case conferencing between inpatient and ambulatory case managers for discharge planning was attempted but limited due to staffing shortages. To address this, the principal investigator placed ACM referral orders for all participants. Of the 40 referrals placed, 30 patients were eligible (ACM does not follow patients discharged to skilled nursing or long-term care facilities). ACM successfully reached 12 patients (40%) and provided 29 distinct services through community-based organizations (Table 2 and Table 3).

**Table 2.** Interventions and outcomes of people who inject drugs (PWID), experiencehomelessness (PEH), or both (PEH and PWID)

	Total N = 42 (%)	PEH or both N = 30 (%)	PWID N = 12 (%)
Addiction medicine consult	32 (76)	22 (73)	10 (83)
Ambulatory case management referral	40 (95)	28 (93)	12 (100)
Ambulatory case management follow-up^*^			
Yes	12 (29)	5 (17)	7 (58)
Unable to reach patient	18 (43)	13 (43)	5 (42)
Discharge medication cost inquiry	11 (26)	8 (27)	3 (25)
Meds-to-bed program	15 (36)	8 (27)	7 (58)
Discharge disposition			
Home (friend, relative)	19 (45)	11 (37)	8 (67)
Self-discharged	12 (29)	8 (27)	4 (33)
Subacute rehab/skilled nursing facility	6 (14)	6 (20)	0
Street	3 (7)	3 (10)	NA
Other	2 (5)	2 (7)	0
Infectious disease follow-up appointment	24 (57)	16 (53)	8 (67)
Patient followed-up	5 (12)	4 (13)	1 (8)
Completed prescribed antibiotic course	18 (43)	10 (33)	8 (67)
Emergency department visits within 90 days	12 (29)	11 (37)	1 (8)
Readmitted within 90 days after discharge	18 (43)	13 (43)	5 (42)
Readmitted for infection	10 (56)	7 (54)	3 (60)
Clinical outcomes at 90 days			
Clinical cure	18 (43)	13 (43)	5 (42)
Progression/relapse	16 (38)	11 (37)	5 (42)
Deceased	2 (5)	2 (7)	0
Unknown	6 (14)	4 (13)	2 (17)

* Six patients were either transferred or discharged to a facility or were deceased and did not qualify for ambulatory case management referral.

**Table 3.** Ambulatory case management services offered to and accepted by patients experiencing homelessness (PEH) and people who inject drugs (PWID)

Services patients accepted	Total N = 10 (%)	PEH N = 3 (%)	PWID N = 7 (%)
Antibiotic education (ie, importance of taking the antibiotic as directed and completing the course and advocating for connection with the provider if side effects arise) to maintain adherence	8 (80)	2 (67)	6 (86)
Healthcare navigation (ie, guidance with Medicaid, primary care provider, follow-up appointments, and home health care)	6 (60)	1 (33)	5 (71)
Transportation services	5 (50)	1 (33)	4 (57)
Housing resources	2 (20)	2 (66)	0
Food pantry resources	2 (20)	0	2 (29)
Job assistance	1 (10)	0	1 (14)

Numerous individual and systemic barriers were identified after formal evaluation with the investigative group, including limited staffing and resources, restricted placement options, unstable housing, and difficulty maintaining contact postdischarge (Table 4). Self-directed discharge occurred in 12 patients (29%), and 22 patients (52%) were discharged home or to the street; only 14% were discharged to a facility.

**Table 4.** Major challenges and barriers

Identification of study participants	Limited z-code usage Documentation in electronic medical record not standardized Delayed identification of eligible patients affected timely execution of study quality improvement processes
Patient barriers	Patient loss to follow-up and non-adherence Many patients lacked access to a working phone making communication difficult after discharge Patients lacked transportation resulting in missed follow-up appointments High healthcare utilization High behavioral health/addiction medicine needs
Structural and social determinants of health barriers	Limited staff capacity to meet needs of all patients (especially case managers) Limited placement options after discharge Lack of affordable housing and supportive housing Limited transportation access Limited behavioral health resources Insurance challenges with accessing some medications, particularly Suboxone

Of the 24 patients (57%) with scheduled infectious diseases follow-up appointments, only 5 (21%) attended. ED visits were frequent, with over one-third of patients readmitted within 90 days due to infection-related complications associated with antibiotic non-adherence. Among the 10 readmitted patients, five (50%) had self-directed discharges with persistent infection; three (30%) were readmitted for disease progression or relapse, including one requiring surgical intervention; and two (20%) had persistent infection. Among those who received antibiotics, 18 (43%) patients achieved clinical cure, and 16 (38%) experienced disease progression within 90 days. Outcomes were unknown for 6 (14%) patients.

## Discussion

This study highlights the complex clinical and social needs of PWID and PEH hospitalized with complicated infections. Despite coordinated multidisciplinary efforts and significant resource utilization, patients had a prolonged hospital length of stay and were commonly readmitted. Several systemic and individual-level barriers were identified, including limited staff capacity, restricted discharge placement options, unstable housing, and difficulty maintaining contact postdischarge. These barriers likely contributed to suboptimal treatment adherence, where less than half of patients achieved clinical cure, and more than one-third experienced disease progression within 90 days.

PWID and PEH hospitalization courses are notably more resource-intensive, marked by higher costs, mortality, and complex postdischarge needs. Specific care barriers, such as access to harm reduction, addiction medicine, and mental health services, and logistical challenges have been described elsewhere.^34^ In the present study, despite engaging addiction medicine, street medicine, and case management throughout the continuum of care, outcomes remained poor, where frequent loss to follow-up, disease progression, clinical failure, ED revisits, and readmissions were observed. Unfortunately, these findings are consistent with other implementation science data evaluating patient-level interventions in hospitalized PWID and PEH. A systematic literature review performed by James et al. evaluated the impact of interventions published in 31 studies that were focused on patients primarily with SUD, including PEH.^35^ Most interventions evaluated led to improvements in patient care when considering multiple outcome types; however, 15.4% of studies evaluated showed no significant difference in outcome after intervention.^35^ The authors concluded that long-term outcomes are not routinely evaluated, making evaluation challenging, but that successful interventions focus on “1) the identification and treatment of SUD in hospital settings; 2) the delivery of evidence-based SUD treatment in the hospital setting; and 3) functional connections to community resources and social supports,” which the present study attempted to address. Similar challenges to the context and interpretation of successful interventions for PEH have been described.^36^ Overall, the results of the present study highlight the urgent need for pragmatic and sustainable strategies that reduce inpatient burden, while strengthening continuity of care after discharge.

Some of the anticipated barriers to success are undoubtedly related to transition of care in PWID and PEH, despite an intensified resource allocation to these services. OPAT offers a pathway to earlier discharge and improved engagement in substance use treatment.^3,6^ Studies suggest that PWID discharged home may have better antibiotic completion rates and lower substance use compared to those discharged to skilled nursing facilities.^3^ However, access to OPAT remains limited due to social barriers, including higher rates of loss to follow-up and self-discharge, and stigma-driven concerns about catheter misuse and infection despite evidence showing low rates of catheter tampering and comparable outcomes to non-PWID populations.^3,17,18^

Early transition to oral antibiotics offers a practical alternative to OPAT for PWID and PEH, who frequently face rejection from postacute care facilities and lower treatment completion rates.^3^ In this study, over half of patients were discharged on oral agents. However, less than half of eligible patients received their medications through the Meds-to-Beds Program due to resource constraints, and pharmacy pickup rates remained low. These findings highlight the need for more reliable medication delivery strategies. Additionally, long-acting lipoglycopeptides, such as dalbavancin, used in 17% of patients, may offer a promising option for simplifying regimens and improving adherence in at-risk populations.^3,17,18^

Despite the many challenges, the project demonstrated meaningful successes. The involvement of an interdisciplinary team, including infectious diseases, clinical pharmacy, addiction medicine, case management, and population health, was essential in addressing the multifaceted needs of this vulnerable population. Pharmacy-led interventions were particularly impactful. Tailored antibiotic education and the integration of the SDOH screening wheel into the pharmacist EHR view improved awareness and responsiveness to patient needs.

The introduction of the Street Medicine Detroit consult order marked a significant advancement in this study. Implemented midway through, it enabled targeted outreach to unsheltered PEH, helping to address gaps left by limitations in ACM follow-up. By engaging patients during hospitalization, the Street Medicine team promoted continuity of care after discharge, effectively bridging a critical gap in service delivery. This community-based healthcare model delivers medical and social services directly to PEH, meeting them where they are, whether on the streets, in encampments, or in shelters. Operating multiple times per week, teams are equipped with vans stocked with essential supplies, including medications, food, hygiene items, and wound care materials. In addition to clinical support, they assist with housing, identification, insurance, and other vital services. Street Medicine serves as a vital link to formal healthcare systems, helping patients overcome barriers to accessing health care and reducing stigma through compassionate, patient-centered care.

Despite efforts to coordinate discharge planning through integrated case conferencing, staffing shortages limited its effectiveness. Among the eligible patients, ACM successfully reached only 40%, underscoring the need for improved contact strategies and resource allocation. For those reached, ACM provided distinct services, demonstrating the potential impact of targeted SDOH interventions.

Although we acknowledge the significant structural, cultural, and operational challenges associated with delivering effective care to PWID and PEH, our findings highlight several actionable strategies that health systems can adopt to improve equity and outcomes in these populations. To address stigma and disengagement, staff training in culturally sensitive care and patient-centered language is essential. Electronic medical records can be optimized through the incorporation of Z-codes and clinical alerts to improve identification of patients with social risk factors and facilitate timely multidisciplinary consultation, mitigating gaps in recognition and follow-up. Expanding SDOH screening across clinical roles and integrating results into electronic workflows may reduce reliance on individual clinician discretion and promote more consistent intervention. In addition, implementing a Community Information Exchange or similar platform can help overcome fragmentation between health systems and community-based organizations by enabling automated, closed-loop referrals to address housing, substance use treatment, and other social needs more efficiently.^37,38^ Finally, qualitative research is needed to better understand patient perspectives and the drivers of self-directed discharge, which may inform more patient-centered and acceptable care models. Collectively, these strategies provide practical approaches for health systems to address known barriers, improve care delivery for PWID and PEH, and align with emerging regulatory expectations focused on SDOH and health equity.

This study has several limitations. As a single-center pilot QI initiative with a small sample size, findings should be interpreted cautiously. Larger, multi-center studies are needed to assess the effectiveness and scalability of multi-component interventions for improving outcomes among PWID and PEH.

## Conclusion

Improving health outcomes for PWID and PEH hospitalized with complicated infections requires a coordinated, interdisciplinary approach that addresses both clinical and SDOH. Despite significant barriers and suboptimal outcomes, targeted interventions such as pharmacy-led education, SDOH screening integration, and the implementation of Street Medicine consults demonstrated meaningful impact.

## Data Availability

None.
